# Risk Factors for and Seroprevalence of Tickborne Zoonotic Diseases among Livestock Owners, Kazakhstan

**DOI:** 10.3201/eid2601.190220

**Published:** 2020-01

**Authors:** Jennifer R. Head, Yekaterina Bumburidi, Gulfaira Mirzabekova, Kumysbek Rakhimov, Marat Dzhumankulov, Stephanie J. Salyer, Barbara Knust, Dmitriy Berezovskiy, Mariyakul Kulatayeva, Serik Zhetibaev, Trevor Shoemaker, William L. Nicholson, Daphne Moffett

**Affiliations:** Association of Schools and Programs for Public Health, Washington, DC, USA (J.R. Head);; Public Health Institute, San Francisco, California, USA (J.R. Head);; Centers for Disease Control and Prevention, Atlanta, Georgia, USA (J.R. Head, S.J. Salyer, B. Knust, T. Shoemaker, W.L. Nicholson);; Centers for Disease Control and Prevention, Almaty, Kazakhstan (Y. Bumburidi, D. Berezovskiy, D. Moffett);; Zhambyl Oblast Public Health Protection Department, Taraz, Kazakhstan (G. Mirzabekova, K. Rakhimov);; Zhambyl Oblast Health Department, Taraz (M. Dzhumankulov);; Zhambyl Oblast Sanitary Epidemiology Expertise Center, Taraz (M. Kulatayeva, S. Zhetibaev)

**Keywords:** Crimean-Congo hemorrhagic fever, Q fever, Lyme disease, *Coxiella burnetii*, *Borrelia burgdorferi*, tickborne infections, vector-borne infections, zoonoses, One Health, livestock, Kazakhstan, bacteria, viruses

## Abstract

Crimean-Congo hemorrhagic fever (CCHF), Q fever, and Lyme disease are endemic to southern Kazakhstan, but population-based serosurveys are lacking. We assessed risk factors and seroprevalence of these zoonoses and conducted surveys for CCHF-related knowledge, attitudes, and practices in the Zhambyl region of Kazakhstan. Weighted seroprevalence for CCHF among all participants was 1.2%, increasing to 3.4% in villages with a known history of CCHF circulation. Weighted seroprevalence was 2.4% for Lyme disease and 1.3% for Q fever. We found evidence of CCHF virus circulation in areas not known to harbor the virus. We noted that activities that put persons at high risk for zoonotic or tickborne disease also were risk factors for seropositivity. However, recognition of the role of livestock in disease transmission and use of personal protective equipment when performing high-risk activities were low among participants.

Zoonoses account for 61% of human infectious diseases and 75% of emerging pathogens (*1*). Zoonotic diseases pass from animals to humans through direct contact with animals, inhalation of infectious aerosols, consumption of contaminated animal products, or a bite from a vector, such as a tick (*2*). Global incidence of tickborne diseases is increasing and expected to continue rising (*3*). Given changes in ecologic factors, such as climate and land use, tickborne diseases have emerged in new areas during the past 3 decades, and the incidence of endemic tickborne pathogens has increased (*4*). Vectorborne infections were responsible for ≈28.8% of emerging infectious diseases during 1990–2000 (*5*). 

Crimean-Congo hemorrhagic fever (CCHF), Q fever, and Lyme disease are widespread zoonotic diseases that cause a range of illness and death in humans. CCHF, caused by Crimean-Congo hemorrhagic fever virus (CCHFV), an RNA virus of the family *Nairoviridae*, is highly fatal (*6*). The virus is maintained through an enzootic cycle involving mammals, ticks, and humans, and is transmitted to humans through contact with viremic livestock or infected ticks. CCHF is endemic to Africa, Asia, eastern and southern Europe, and central Asia (*7*). Q fever is caused by the bacterium *Coxiella burnetii*, which infects many vertebrates, but ruminant livestock are thought to be its primary reservoir. Transmission to humans most commonly occurs from inhalation of dust contaminated with urine, feces, milk, or birth products from infected animals (*8*). Q fever has been identified in most countries (*8*). Severe cases can result in pneumonia or hepatitis in humans, and ≈5% of infections become chronic (*9*). Lyme disease is caused by *Borrelia burgdorferi*, a bacterium transmitted to humans through the bite of *Ixodes* ticks. Untreated Lyme infection can disseminate and spread to the joints, heart, and nervous system. Lyme disease is the most commonly reported arthropodborne disease in North America and is prevalent throughout central Europe, particularly Germany, Austria, and Slovenia (*10*,*11*). Lyme disease is the sixth most commonly reported notifiable infectious disease in the United States (https://www.cdc.gov/lyme). In addition, incidence of Lyme disease and the range of tick vectors have been increasing in Europe and Asia (*10*,*11*), where Lyme disease is found in western Russia, Mongolia, northeast China, and Japan.

CCHF, Q fever, and Lyme disease are endemic to the southern Kazakhstan region of Zhambyl, but their true burden is largely unknown because few serologic surveys have been conducted in Kazakhstan and central Asia. The Zhambyl region covers >55,000 km^2^ and has a population of ≈1.2 million. The region is characterized by diverse ecology, containing both desert steppes and mountainous pastures, and elevations of 213–4,115 m. The region has 363 villages and 4 cities. Livelihoods are largely pastoral or agricultural, and common occupations involve a high degree of animal contact, placing humans at increased risk for zoonotic infections.

Among the 3 diseases, only CCHF is a reportable disease in Kazakhstan. During 2000–2013, the Zhambyl region had 73 reported human CCHF cases, the second highest case-count among regions in Kazakhstan (*12*). However, data on human prevalence of CCHF in Kazakhstan are limited to reported clinical cases, even though studies show >80% of infections are subclinical (*13*). Although Q fever was detected in Kazakhstan in the 1950s, the lack of surveillance or serologic studies obscure our understanding of Q fever or Lyme disease incidence in the population (*14*). Quantifying seroprevalence of these diseases in humans can help identify areas of pathogen circulation and areas where humans could be infected.

For this study, we aimed to determine the seroprevalence of antibodies against CCHFV, *C. burnetii*, and *B. burgdorferi* in humans who interact with livestock in the Zhambyl region. In addition, we sought to assess the population’s knowledge of risk factors for disease transmission and how frequently they engage in activities that increase or reduce risk for infection.

## Methods

In June 2017, we conducted a knowledge, attitudes, and practices and risk factor survey (KAP/risk factor survey), along with serosurveys for CCHF, Q fever, and Lyme disease, in 30 rural villages in the Zhambyl region. Participants could enroll in the KAP/risk factor survey, the serosurvey, or both. Eligible participants were >18 years of age, residents of the village for >2 months, and residents of a household containing a sheep or cow of >1 year of age.

### Sample Size

Sample selection was based on concern about CCHF as a nationally reportable disease. We conducted surveys in households in which sheep and cattle serosurveys simultaneously were conducted; sample size calculations were based on expected seroprevalence of sheep and cattle. We calculated a target sample size of 561 households with sheep and 473 households with cattle. We based the sample size on an α of 0.05, power of 80%, a design effect of 2, and an expected response rate of 90%. We assumed CCHF seroprevalence of 24% in sheep and 19% in cattle, on the basis of a meta-analysis of previous serosurveys (*15*).

### Participant Selection

We stratified the 363 villages in the region by known (CCHF-endemic) or unknown (non–CCHF-endemic) recent circulation of CCHF. We defined recent circulation as 1 confirmed human case reported in hospital-based surveillance from Zhambyl Oblast Health Department (Taraz, Kazakhstan) or 1 CCHF-positive tick confirmed in the previous 5 years and reported in annual tick surveillance data from the Ministry of Agriculture of Kazakhstan. We identified 66 (18.2%) villages that met the definition for having known, recent CCHF circulation.

We selected 15 villages from each stratum; probability of selection was proportional to the number of sheep and cattle in the village. We obtained livestock counts from reports by village veterinarians to the Ministry of Agriculture. Elevation of the 30 villages was 220–2,590 m (mean 781 m, median 488 m). Mean elevation was 513 m for villages with known CCHF circulation and 1,049 m for villages without known circulation.

Local veterinarians provided information on livestock-owning households in each village. To verify, data collectors conducted a census of 5 villages and mapped households containing sheep or cattle. The veterinarian registry was accurate except for 2 instances in which the household recently had sold animals. Survey teams randomly selected 35 households from these registries and 1 adult per household for study participation.

#### KAP/Risk Factor Survey

We adapted our KAP/risk factor survey from one conducted in Georgia during a 2014 CCHF outbreak (*16*). We translated the survey into Russian and Kazakh, the 2 most common languages in the region. Survey teams pilot tested the questionnaire in an eligible village not selected for the study.

The survey team administered the questionnaire verbally at each respondent’s residence. Survey questions covered demographics; occupations; history of animal and tick interactions; illness in the previous 4 months or fever and hemorrhaging; and knowledge of CCHF transmission routes, symptoms, and risk factors. The survey did not contain questions specific for Lyme disease or Q fever.

#### Serosurvey

After answering the questionnaire, respondents were asked to go to their local health clinic to provide a blood sample on the same day. Each village had a health clinic within walking distance of participants. Nurses drew 5 mL of blood from each participant and stored it in a serum separator tube. Blood samples were kept on ice, centrifuged within 6 h, and transported within 24 h to the Zhambyl Regional Laboratory for Especially Dangerous Pathogens in Taraz, where laboratorians aliquoted serum into 4 samples/participant and stored serum at −20°C until analysis.

Laboratorians analyzed samples for evidence of recent CCHF exposure, indicated by presence of IgM, by using VectoCrimean-CCHF-IgM Kits (Vector-Best, https://vector-best.ru) and for evidence of past CCHF exposure, indicated by IgG, by using VectoCrimean-CHF-IgG Kits (Vector-Best). Laboratorians assessed past exposure to *C. burnetii*, indicated by presence of IgG*,* using ELISA-Anti-Q Kit No. 1 (Pasteur Institute of Epidemiology and Microbiology, http://www.pasteur-nii.spb.ru), and exposure to *Borellia* spp., indicated by presence of IgG against *B. afzelii*, *B. garinii*, or *B. burgdorferi*, by using LymeBest-IgG Test Kits (Vector-Best). All testing was performed with commercially available ELISA kits, according to manufacturer instructions (*17*,*18*).

### Data Analysis

We analyzed data by using R version 3.4.3 (*19*). We weighted results for each participant by calculating the inverse probability of selection and applying a poststratification adjustment to each stratum to account for nonresponses. We stratified KAP/risk factor answers specific to CCHF according to whether the health department recognized the village as having known, recent history of CCHF. We used χ^2^ test in bivariate analysis to compare frequencies between these 2 strata. We used logistic regression models to test associations between risk factors and seropositivity. We defined risk for zoonotic or tickborne disease as participation in >1 of the following activities: handling ticks with bare hands; working with livestock; working in a healthcare setting; being a veterinarian; or herding, birthing, shearing, slaughtering, or milking animals.

### Ethics Review

Each participant provided written, informed consent. No personal identifying information was collected. The Institutional Review Board in Almaty, Kazakhstan, through the Committee for Public Health Protection, approved the study. The protocol was reviewed according to the US Centers for Disease Control and Prevention human subjects review procedures, which determined the agency was not engaged in the study because the Zhambyl Departments of Health and Agriculture owned and collected the data.

## Results

### KAP/Risk Factor Survey

We selected 969 households; 948 persons completed surveys, a 98% response rate. Reasons for nonresponse included 4 households that were not visited, 2 that were abandoned, and 1 that was not found. In addition, 12 persons did not consent: 4 did not want to participate in the serosurvey, 1 did not have time, 1 distrusted the data team, and 6 gave no reason. Further, 2 persons were excluded from analysis because information on their village of residence was missing and they could not be analyzed according to survey design.

Respondents’ median age was 46 (range 19–90) years; 54% were male (Table 1). Most (66.7%) were native to Kazakhstan. The most frequently reported occupations were taking care of the home (23.0%) and farming or herding (20.8%).

**Table 1:** Demographic characteristics of study population in survey of Crimean-Congo hemorrhagic fever, Kazakhstan

Patient characteristics	Median (IQR)	Range
Age, y	46 (36–56)	19–90
Household size	6.1 (4.6–8.4)	2.7–21.6
Land owned, ha	0.18 (0.12–0.25)	0.004–776
Land rented, ha	0.20 (0.14–0.90)	0.024–776
No. animals owned		
Ovine	15.0 (3.0–35.0)	0–1,320
Bovine	2.0 (1.0–5.0)	0–141
Poultry	0 (0–8.0)	0–80
Equine	0 (0–1.0)	0–100
	No. participants	% Participants (95% CI)
Sex		
M	509	56.1 (50.5–61.5)
F	437	43.9 (38.5–49.5)
Country of origin		
Kazakhstan	733	66.7 (44.0–85.9)
Russia	73	10.5 (4.4–23.0)
Turkey	45	4.4 (1.9–9.2)
Kyrgyzstan	3	0.6 (0.2–1.9)
Uzbekistan	3	0.4 (0.1–2.1)
Other	89	15.7 (4.0–45.7)
Occupation		
Farmer, herder, animal tender	163	20.8 (10.1–38.1)
Gardener, fieldworker	50	3.1 (1.3–7.4)
Butcher	1	0.001 (0–0.01)
Healthcare worker	21	2.5 (1.5–4.1)
Veterinarian	15	1.5 (0.6–4.1)
Office, indoor worker	153	14.0 (9.0–21.1)
Family or home caretaker	179	23.0 (18.4–28.5)
Student	10	1.1 (0.4–3.1)
Retired	147	9.8 (6.3–14.9)
Unemployed	105	14.3 (5.8–31.0)
Other	101	9.9 (3.4–25.6)
Education level		
None	12	0.1 (0.03–0.5)
Elementary school	9	0.8 (0.3–2.0)
Middle school	437	44.1 (33.9–54.9)
High school	159	11.5 (7.5–17.2)
Vocational school	71	4.1 (1.7–9.5)
College	251	38.9 (28.3–50.8)
Monthly income, US $		
<60	43	4.0 (1.3–11.4)
61–150	373	39.1 (24.6–55.8)
151–300	257	26.8 (20.1–34.6)
301–450	34	0.8 (0.2–3.0)
451–600	9	0.5 (0.1–2.1)
>600	7	0.2 (0.04–1.1)
Unknown, refused to answer	222	28.6 (12.6–52.6)

Of respondents, 64.4% (95% CI 50.9%–75.8%) reported participating in >1 activity putting them at elevated risk for zoonotic or tickborne disease during their lives; 55.4% (95% CI 42.8%–67.3%) reported doing so in the previous 4 months (Table 2). Of high-risk activities, butchering or handling raw meat (36.4%) and shearing (26.0%) or herding (25.8%) animals were most common. Of respondents, 139 (22%) who birthed animals in the previous 4 months and 222 (47.4%) who slaughtered an animal in the previous 4 months wore no personal protective equipment (PPE). Few respondents reported tick bites (Table 3), but >85% said ticks were a major problem (Table 4). Most respondents (93.6%) reported killing ticks with an object; only 0.5% reported killing ticks with bare hands. Most (94.0%) used pesticide to prevent ticks on animals.

**Table 2:** Participation in activities putting them at high risk for tickborne zoonotic diseases among respondents in survey of Crimean-Congo hemorrhagic fever, Kazakhstan*

Activities	No. respondents	% Respondents (95% CI)
Herding animals		
Ever	297	17.4 (8.4–32.5)
Within the previous 4 mo	190	25.8 (14.3–42.2)
Assisting with animal births		
Ever	226	11.3 (6.8–18.3)
Within the previous 4 mo	140	5.9 (3.5–9.9)
Shearing animals		
Ever	331	26.0 (19.7–33.4)
Within the previous 4 mo	223	17.0 (12.9–22.1)
Milking animals		
Ever	316	23.2 (16.3–31.9)
Within the previous 4 mo	251	18.9 (12.8–27.0)
Slaughtering animals		
Ever	292	25.4 (15.8–38.1)
Within the previous 4 mo	229	20.4 (12.0–32.4)
Butchering or handling raw meat		
Ever	351	36.4 (28.4–45.2)
Within the previous 4 mo	296	30.7 (22.7–40.0)
Eating raw meat		
Ever	8	0.5 (0.1–1.9)
Within the previous 4 mo	0	–
Handling ticks with bare hands		
Ever	61	3.5 (1.1–10.3)
Within the previous 4 mo	27	2.0 (0.4–8.4)
Working in a healthcare setting		
Ever	5	0.3 (0.1–0.9)
Within the previous 4 mo	3	0.2 (0–0.8)
Working in a garden†		
Ever	175	14.5 (7.6–27.4)
Within the previous 4 mo	150	12.4 (6.5–22.6)
Consuming unpasteurized milk or dairy products‡		
Ever	8	1.0 (0.4–2.2)
Within the previous 4 mo	0	–
Participated in >1 high-risk activity		
Ever	683	64.4 (50.9–75.8)
Within the previous 4 mo	580	55.4 (42.8–67.3)
Use of personal protective equipment		
Assisting with animal births, n = 139†		
Gloves	73	55.2 (35.8–73.1)
Gown	43	55.1 (30.3–77.6)
Boots	21	30.0 (11.8–58.0)
Glasses	3	12.7 (2.0–51.5)
None	46	20.4 (11.0–34.7)
Shearing animals, n = 222†		
Gloves	172	83.6 (71.7–91.2)
Gown	119	73.9 (55.9–86.4)
Boots	59	20.6 (12.2–32.6)
Glasses	4	2.9 (0.7–11.4)
None	21	5.2 (1.7–15.2)
Milking animals, n = 250†		
Gloves	26	15.5 (5.5–36.8)
Gown	178	81.6 (60.6–92.7)
Boots	12	5.7 (2.2–14.1)
None	71	17.5 (6.4–39.5)
Slaughtering animals, n = 229†		
Gloves	36	23.3 (10.5–44.1)
Gown	91	49.1 (33.6–64.8)
Boots	16	5.2 (2.0–12.9)
Glasses	1	1.6 (0.2–9.4)
None	129	47.4 (29.8–65.7)
*Percentages weighted by calculating the inverse probability of selection and applying a post-stratification adjustment to each stratum to account for nonresponses. †>1 response possible. ‡Not considered a high-risk activity.		

**Table 3:** Interactions with ticks among respondents in survey of Crimean-Congo hemorrhagic fever, Kazakhstan*

Human–tick interactions	No. respondents	% Respondents (95% CI)
Had a tick bite†	17	1.0 (0.3–3.3)
Handled tick with bare hands†	61	3.5 (1.1–10.3)
Method of tick disposal after bare hand removal, n = 27		
Threw it out	1	3.2 (0.3–29.3)
Killed with bare hands†	1	0.5 (0–5.9)
Killed with object	16	93.6 (69.2–99.0)
Burned it	10	3.5 (0.6–18.8)
Number of tick bites in previous 4 mo	0	0
Method of human tick bite prevention‡		
None	133	9.3 (3.9–20.8)
Long, layered clothing	694	68.8 (55.2–79.9)
Gloves	588	73.1 (60.5–82.9)
Pesticides in environment	267	13.8 (7.9–22.9)
Insect repellent on self, clothing	155	17.7 (10.0–29.3)
Avoiding woody areas	133	12.2 (4.1–31.0)
Avoiding unnecessary animal contact	111	13.9 (5.0–33.3)
Animal–tick interactions		
Found ticks on livestock	486	29.7 (19.6–42.3)
Primary method used to remove ticks on livestock		
Bare hands†	12	4.3 (1.2–15.0)
Gloved hands	95	29.8 (15.9–48.7)
With an object	291	51.7 (34.0–69.0)
Go to a clinic	15	3.3 (1.2–8.7)
Pour liquid mixture on animal	32	3.0 (1.2–7.1)
Burn the tick	6	0.7 (0.2–2.2)
Leave the tick	31	6.8 (2.6–16.3)
Use tick medication for animals	905	94.0 (76.0–98.8)

*Percentage weighted by calculating the inverse probability of selection and applying a poststratification adjustment to each stratum to account for nonresponses. †High-risk tick interaction. ‡>1 response possible.

**Table 4:** Comparison of respondent attitudes between CCHF-endemic villages and non–CCHF-endemic villages in survey of Crimean-Congo hemorrhagic fever, Kazakhstan*

Attitudes	CCHF-endemic, n = 442			Non–CCHF-endemic, n = 506		p value
	No. respondents†	% Respondents (95% CI)		No. respondents†	% Respondents (95% CI)	
Among all persons						
Ticks are a problem in the community						0.05
Major problem	410	95.3 (89.9–97.9)		408	86.6 (67.8–95.2)	
Minor problem	4	0.7 (0.2–3.0)		13	2.1 (0.8–5.6)	
Not a problem	3	0.6 (0.1–3.1)		52	5.0 (1.0–21.9)	
Don’t know	23	3.4 (1.2–9.0)		33	6.4 (2.7–14.4)	
People in my community frequently get bitten by ticks						0.74
Often	245	49.0 (19.4–79.3)		187	33.5 (12.2–64.7)	
Occasionally	24	7.4 (1.3–32.2)		94	13.3 (5.4–29.4)	
Rarely	149	40.4 (17.7–68.1)		202	50.2 (20.7–79.5)	
Don’t know	22	3.2 (1.4–7.2)		23	3.0 (0.7–12.1)	
Among persons who have heard of CCHF	n = 420			n = 371		
CCHF is a problem in the community						0.12
Major problem	401	96.2 (90.0–98.6)		326	93.7 (82.7–97.9)	
Minor problem	3	0.7 (0.2–3.1)		9	1.9 (0.5–6.6)	
Not a problem	1	0.1 (0–0.5)		26	2.7 (0.5–13.5)	
Don’t know	15	3.0 (1.1–8.4)		10	1.7 (0.5–6.1)	
CCHF is something I should be worried about						0.01
Very worried	371	86.1 (72.5–93.5)		317	93.6 (83.5–97.7)	
Somewhat worried	40	11.5 (4.2–27.8)		19	2.6 (0.9–7.4)	
Not worried	1	0.02 (0–0.2)		25	2.5 (0.4–13.9)	
Don’t know	8	2.4 (0.4–12.4)		10	1.2 (0.2–7.1)	
I can protect myself from CCHF						<0.01
Yes	380	90.5 (82.5–95.0)		191	52.5 (33.6–70.6)	
No	4	0.7 (0.2–3.2)		100	22.7 (8.3–48.8)	
Don’t know	36	8.9 (4.2–17.9)		80	24.8 (12.6–43.0)	
I would welcome a CCHF survivor into my community	379	89.2 (79.7–94.5)		348	94.2 (87.9–97.4)	0.17

*CCHF, Crimean Congo hemorrhagic fever. †Percentage weighted by calculating the inverse probability of selection and applying a poststratification adjustment to each stratum to account for nonresponses.

Participants from CCHF-endemic villages had a higher knowledge of CCHF, likely because the health department provided education in these villages (Table 5). Most respondents (95.6%, 95% CI 93.8%–99.9%) in CCHF-endemic villages had heard of CCHF, compared with only 71.3% (95% CI 61.7%–79.3%) in non–CCHF-endemic villages (Table 5). Information from healthcare workers, pamphlets, and village meetings were common ways participants learned about CCHF. In addition, 95.8% (95% CI 89.8%–98.3%) of respondents in CCHF-endemic villages who knew about CCHF could recognize >1 high-risk activity, compared with 75.9% (95% CI 49.1%–91.1%) in non–CCHF-endemic villages. Most recognized tick bites as a mode of transmission, and >10% in CCHF-endemic villages recognized animal blood as a potential mode of transmission. Despite a lower disease knowledge in non–CCHF-endemic villages, respondents thought CCHF was a major problem (Table 4), but only 52.5% felt prepared to protect themselves from CCHF, compared with 90.5% from CCHF-endemic villages. 

**Table 5:** Comparison of participant knowledge of Crimean-Congo hemorrhagic fever between CCHF-endemic villages and non–CCHF-endemic villages, Kazakhstan*

Information about CCHF	CCHF-endemic villages, n = 442		Non–CCHF-endemic villages, n = 506	p value

No. participants†	% Participants (95% CI)	No. participants†	% Participants (95% CI)
Heard of CCHF	420	95.6 (93.8–99.9)		371	2.0 (61.7–79.3)	<0.001
Source*	n = 420			n = 371		
Village, community meetings	262	62.1 (28.0–87.3)		110	46.9 (16.2–80.1)	
School	77	18.9 (8.8–35.9)		25	4.1 (1.5–11.1)	
Television	196	60.5 (42.7–75.9)		333	87.4 (77.9–93.2)	
Radio	34	8.1 (3.7–16.7)		10	2.1 (0.7–6.1)	
Newspaper, magazine	153	43.6 (28.7–59.6)		117	32.7 (25.1–41.3)	
Pamphlets	240	72.5 (51.2–86.9)		248	79.5 (61.7–90.3)	
Posters	225	69.6 (43.8–87.0)		185	66.1 (45.9–81.8)	
Education campaign	18	4.0 (1.3–11.4)		0	0	
Training course	3	90.6 (0.1–3.4)		7	2.8 (0.9–8.6)	
Healthcare worker	329	89.0 (78.8–94.6)		175	62.0 (31.2–85.5)	
Know someone with CCHF	12	3.2 (1.2–8.5)		30	4.7 (3.1–7.0)	
Other, friend, family member	8	2.2 (0.7–6.9)		1	1.0 (0.2–5.5)	
Transmission						
Tick bite	415	99.6 (98.5–99.9)		367	99.4 (97.8–99.9)	
Mosquito bite	0	0		0	0	
Flea bite	5	0.05 (0–0.4)		3	0.4 (0.1–2.7)	
Crushing tick with bare hands	218	53.8 (21.4–83.3)		96	31.3 (15.5–53.0)	
Contact with blood from an infected animal	50	9.10 (3.5–21.7)		91	21.0 (7.1–48.1)	
Contact with birthing tissue of infected animal	16	2.0 (0.6–6.4)		66	16.1 (5.1–40.7)	
Eating or touching raw meat of infected animal	12	1.3 (0.4–4.5)		49	12.4 (4.1–32.0)	
Contact with blood from infected human	186	41.6 (28.2–56.3)		183	45.0 (30.2–60.9)	
Contact with persons sick with CCHF	135	37.8 (19.3–60.6)		204	67.9 (47.3–83.3)	
Unpasteurized milk	7	0.3 (0.1–1.7)		10	1.9 (0.5–7.3)	
Correctly identified >1 transmission route	413	99.6 (98.5–99.9)		367	99.6 (97.8–99.9)	0.90
High-risk activities						
Working with livestock	386	94.7 (86.6–98.0)		168	52.8 (30.4–74.1)	
Gardening or fieldwork	144	50.1 (27.5–72.7)		89	30.4 (17.0–48.2)	
Being in a woody area	115	30.6 (13.0–56.5)		152	55.1 (33.6–74.9)	
Crushing ticks with bare hands	261	62.2 (30.8–85.8)		132	39.3 (26.4–53.9)	
Shearing animals	231	53.7 (29.0–76.7)		102	37.2 (18.4–60.7)	
Butchering meat	79	16.7 (7.4–33.6)		50	14.7 (5.0–35.9)	
Working in healthcare	67	14.3 (5.6–31.8)		51	6.4 (4.1–9.7)	
Slaughtering animals	35	7.5 (3.8–14.3)		17	4.1 (1.6–10.2)	
Being a veterinarian	120	28.5 (12.7–52.1)		76	29.1 (11.2–57.2)	
Contact with sick humans	222	55.2 (33.8–74.8)		266	77.8 (66.6–86.1)	
Don’t know	9	0.8 (0.2–3.0)		2	0.04 (0–0.3)	
Correctly identified at >1 high-risk activity	378	95.8 (89.8–98.3)		248	75.9 (49.1–91.1)	0.001
Signs and symptoms in humans						
Fever	398	96.8 (92.7–98.6)		312	93.0 (79.4–97.8)	
Headache	302	74.5 (44.0–91.6)		297	88.7 (71.0–96.2)	
Nausea, vomiting	183	55.8 (36.8–73.2)		86	25.0 (13.8–41.1)	
Diarrhea	75	22.4 (12.4–37.2)		59	20.3 (11.6–33.3)	
Muscle pain	190	48.0 (26.1–70.6)		81	19.3 (8.5–38.1)	
Joint pain	222	59.8 (46.3–72.1)		158	47.6 (32.7–62.9)	
Fatigue	224	66.3 (52.8–77.6)		240	66.0 (51.5–77.9)	
Cough, respiratory involvement	53	12.9 (5.1–29.0)		26	5.6 (1.3–21.6)	
Congestion	110	27.2 (14.6–44.8)		15	4.5 (1.4–13.4)	
Bruising	115	27.7 (15.2–45.1)		59	10.1 (4.4–21.4)	
Bleeding, hemorrhage	251	57.2 (42.6–70.7)		129	26.4 (15.3–41.7)	
Red eyes	55	14.1 (7.5–24.9)		6	0.6 (0.2–2.3)	
Coma	21	4.6 (2.1–10.0)		3	0.3 (0.1–1.7)	
Death	73	17.0 (6.8–36.4)		13	1.2 (0.3–4.7)	
Don’t know	19	1.5 (0.5–4.6)		10	0.7 (0.1–3.9)	
Correctly identified >1 symptom in humans	395	98.0 (94.6–99.3)		346	97.0 (90.6–99.1)	0.57
Signs and symptoms in animals						
None (correct response)	69	20.0 (3.4–64.0)		35	16.0 (2.0–64.3)	0.84
Fever	149	36.5 (10.5–73.9)		203	44.0 (14.9–78.0)	
Aggression, agitation	64	13.3 (4.7–32.6)		130	31.1 (10.3–63.8)	
Vomiting	84	27.2 (7.1–64.6)		30	7.0 (1.3–30.9)	
Lethargy	87	23.0 (6.6–56.0)		151	28.5 (10.2–58.3)	
Abortion, stillbirth	77	20.5 (6.1–50.4)		46	9.8 (2.7–29.9)	
Reduced appetite	75	22.1 (6.3–54.4)		145	33.0 (11.8–64.3)	
Hair loss	60	16.3 (4.6–44.0)		34	7.2 (1.5–28.5)	
Weight loss	55	15.3 (4.2–42.3)		38	6.6 (1.3–26.6)	
Reduced milk production	54	15.7 (4.8–41.0)		26	5.0 (1.3–17.5)	
Respiratory problems	29	7.7 (2.4–21.8)		18	2.2 (0.5–9.4)	
Bleeding, hemorrhage	52	11.1 (3.6–28.9)		47	9.4 (3.2–24.7)	
Eye problems	13	3.1 (0.6–15.2)		3	0.3 (0.04–2.7)	
Death	12	1.3 (0.4–3.8)		2	0.2 (0.04–1.1)	
Don’t know	71	13.8 (5.4–30.7)		2	1.0 (0.2–5.4)	

*>1 response possible in each category. CCHF, Crimean-Congo hemorrhagic fever. †Percentage weighted by calculating the inverse probability of selection and applying a post-stratification adjustment to each stratum to account for nonresponses.

### Serosurveys

Of 948 persons completing the KAP/risk factor survey, 914 (96.4%) submitted blood samples. Of 34 persons who did not participate in the serosurvey, 10 did not show up for a blood draw, 4 did not have time, 2 feared needles, 1 feared consequences of detection, 1 had recent surgery, and 16 reported no reasons. Serum from 914 samples was tested for evidence of CCHF. In addition, 911 samples were tested for Lyme disease, 910 were tested for Q fever, and 4 did not have adequate sample volume for Lyme disease and Q fever testing.

**Figure 1:**
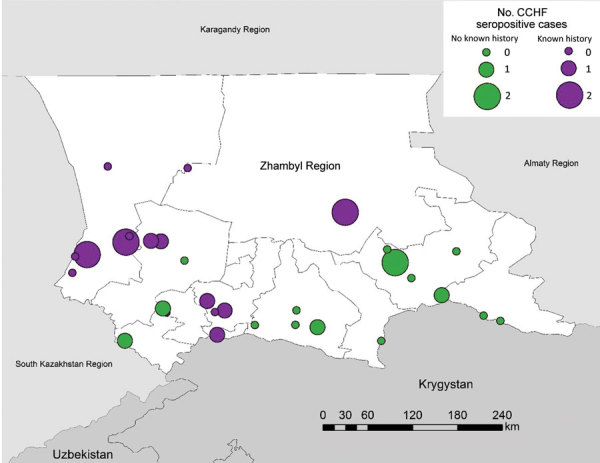
Number of CCHF-seropositive cases in villages included in serologic survey for tickborne diseases, Zhambyl region, Kazakhstan. Circle size denotes the number of IgG antibody–positive serology results indicating past exposure or IgM antibody–positive serology results indicating recent exposure to CCHF. Purple circles indicate that the village had previous known history of CCHF; green circles indicate the village had no known history of CCHF. CCHF, Crimean-Congo hemorrhagic fever.

Of 914 persons tested for CCHFV, 3 were positive for IgM, 12 for IgG, and 2 were positive for both (Table 6). Among livestock owners in the Zhambyl region, weighted CCHFV seroprevalence was 1.2% (95% CI 0.5%–2.7%). In CCHF-endemic villages, seroprevalence was 3.4% (95% CI 1.8%–6.43%), compared with 0.9% (95% CI 0.3%–2.7%) in non–CCHF-endemic villages. We found evidence of recent or past CCHFV exposure in persons from 13/30 (43.3%) villages (Figure 1).

**Table 6:** Characteristics of persons seropositive for 3 tickborne diseases, Kazakhstan*

Characteristics	CCHF (17/914)					Lyme (27/911)					Q fever (11/910)				
	Median	IQR		Range		Median	IQR		Range		Median		IQR		Range
Age, y	54.0	42.0–58.0		21.0–68.0		40.0	32.0–46.5		23.0–76.0		50.0		36.5–56.5		25.0–72.0
Household size	6.1	4.7–6.7		3.9–13.3		5.2	4.1–6.6		2.8–12.4		5.2		4.9–6.2		4.7–13.3
Land ownership, ha	0.15	0.12–0.20		0.01–5.0		0.2	0.13–0.25		0.03–9.0		0.2		0.1–0.3		0–5
Animals owned, no.															
Ovine	7.0	0–20.0		0–200		15.0	6.0–29.5		0–127		13.0		1.0–39.5		0–105
Bovine	2.0	2.0–5.1		0–8.0		2.0	2.0–4.0		0–10		3.0		2.5–4.0		0–12
Poultry	0	0–3.0		0–23		0	0–7.5		0–32		0		0–11		0–34
Equids	0	0		0–2.0		0	0–1.5		0–15		0		0–1.5		0–6
	No. persons		% Persons (95% CI)			No. persons		% Persons (95% CI)			No. persons			% Persons (95% CI)	
Total	17		1.2 (0.5–2.7)			27		2.4 (1.2–4.6)			11			1.3 (0.3–5.0)	
Village type															
CCHF-endemic	11		3.4 (1.7–6.4)			13		2.0 (0.7–5.9)			3			0.1 (0.02–0.9)	
Non–CCHF-endemic	6		0.9 (0.3–2.7)			14		2.5 (1.1–5.2)			8			1.5 (0.4–5.9)	
Sex															
M	10		70.8 (31.9–92.6)			18		70.1 (42.5–88.2)			5			35.4 (26.8–44.9)	
F	7		29.2 (7.4–68.1)			9		29.9 (11.8–57.5)			6			64.6 (55.1–73.1)	
Country of origin															
Kazakhstan	14		70.3 (24.6–94.5)			24		89.1 (42.8–98.9)			7			96.7 (46.8–99.9)	
Russia	2		10.5 (1.6–45.4)			1		0.2 (0–2.3)			4			3.3 (0.1–53.2)	
Turkey	0		0			1		0.1 (0–0.7)			0			0	
Other	1		19.1 (1.7–75.6)			1		10.7 (1.0–57.7)			0			0	
Occupation															
Farmer, herder, animal tender	2		24.2 (3.6–73.5)			5		27.2 (6.0–68.6)			1			1.0 (0–36.6)	
Gardener, fieldworker	0		0			1		0.2 (0–2.3)			0			0	
Healthcare worker	1		3.2 (0.3–28.3)			1		4.4 (0.4–34.2)			1			7.3 (0.1–81.4)	
Veterinarian	0		0			1		0.1 (0–0.7)			0			–	
Office, indoor worker	1		5.4 (0.6–34.2)			7		32.1 (7.0–74.7)			1			7.3 (0.1–81.4)	
Family or household caretaker	3		11.4 (2.9–35.7)			3		0.9 (0.1–7.4)			4			53.7 (17.8–86.1)	
Student	0		0			0		0			0			0	
Retired	5		13.6 (3.8–38.7)			2		10.2 (1.1–54.4)			1			1.3 (0–43.9)	
Unemployed	4		40.0 (9.8–80.3)			4		20.6 (5.1–55.4)			0			0	
Other	1		2.2 (0.2–17.9)			3		4.4 (1.2–14.9)			3			29.5 (14.8–50.2)	
Education level															
None	1		0.02 (0–0.2)			0		0			1			0.5 (0–14.0)	
Elementary school	0		0			0		0			0			0	
Middle school	5		53.9 (18.4–85.8)			10		35.8 (9.6–74.7)			4			87.4 (19.0–99.5)	
High school	3		10.9 (2.6–36.4)			5		4.1 (1.0–15.8)			2			10.2 (0.3–80.4)	
Vocational school	1		0.9 (0.1–8.3)			1		1.1 (0.1–9.2)			1			0.5 (0–14.0)	
College	7		34.3 (8.4–74.8)			11		58.9 (25.0–86.0)			2			1.0 (0–18.9)	
Monthly income, US $															
<60	0		0			1		2.6 (0.2–23.4)			2			1.0 (0–38.9)	
61–150	10		53.7 (17.5–86.3)			9		26.9 (5.9–68.5)			3			31.0 (16.9–49.9)	
151–300	4		35.0 (7.5–78.0)			11		55.2 (20.0–85.9)			1			7.3 (0.1–81.4)	
301–450	1		0.5 (0.1–5.3)			1		0.6 (0.1–5.2)			1			0.3 (0–9.3)	
451–600	0		–			1		0.6 (0.1–5.2)			0			0	
Don’t know, refused	2		10.9 (1.2–55.8)			4		14.0 (2.2–54.2)			3			60.3 (41.6–76.4)	
Length of time in village															
Whole life	15		76.9 (27.0–96.8)			23		76.9 (27.0–96.8)			6			42.2 (14.6–75.7)	
>5 y	1		4.0 (0.3–33.6)			1		4.0 (0.3–33.7)			1			52.5 (16.1–86.5)	
4 mo–5 y	1		19.1 (1.8–75.6)			2		19.1 (1.8–75.6)			3			2.3 (0–60.1)	
2–<4 mo	0		0			0		0			1			2.9 (0–65.7)	
Activities participated in during lifetime															
Herding animals	8		28.9 (10.6–58.1)			10		17.7 (3.9–53.1)			4			34.4 (26.3–43.6)	
Assisting with animal births	6		31.8 (6.7–75.2)			7		5.8 (1.5–20.6)			4			9.1 (0.3–79.0)	
Shearing animals	5		12.6 (3.7–35.2)			13		27.9 (9.7–58.3)			6			35.9 (26.8–46.1)	
Milking animals	8		28.3 (8.0–64.1)			9		22.0 (5.7–56.8)			4			15.6 (0.3–92.8)	
Slaughtering animals	6		35.6 (7.9–78.1)			9		19.5 (4.6–54.6)			5			34.9 (26.5–44.5)	
Butchering, handling raw meat	5		34.7 (7.5–77.9)			11		33.3 (9.8–69.6)			5			30.6 (16.3–49.9)	
Eating raw meat	0		0			0		0			0			0	
Handling ticks with bare hands	0		0			4		1.2 (0.2–7.1)			0			0	
Having tick on body	0		0			0		0			0			0	
Working in healthcare	0		0			1		4.4 (0.4–34.2)			0			0	
Working in garden†	1		2.2 (0.2–17.9)			3		10.0 (1.0–54.6)			3			1.6 (0.1–33.1)	
Consuming unpasteurized dairy†	0		0			0		0			0			0	
>1 high risk activity	15		90.9 (60.3–98.5)			21		53.5 (18.6–85.1)			9			46.2 (14.0–81.9)	
Activities without personal protective equipment‡															
Birthing animals	1		8.3 (0.7–55.0)			2		73.6 (25.4–93.8)			0			0	
Shearing animals	1		6.7 (0.4–54.8)			0		0			0			0	
Milking animals	1		2.3 (0.2–24.0)			2		39.7 (3.6–92.8)			0			0	
Slaughtering animals	4		83.3 (20.9–98.9)			3		14.0 (1.7–61.0)			2			77.3 (42.0–99.6)	
Illness with hemorrhaging and fever, ever	0		0			0		0			1			0.5 (0–14.0)	

*CCHF, Crimean Congo hemorrhagic fever. †Not considered a high-risk activity. ‡No. participants who participated in this activity at any time.

Of the 17 persons seropositive for CCHFV, median age was 54 years; 58% were male (Table 6). No persons reported previous CCHF diagnosis or illness with fever and hemorrhaging in the previous 5 years or a tick bite or handling ticks with bare hands in the previous 4 months. Occupations among the 17 seropositive persons were farmer or herder (n = 2), healthcare worker (n = 1), office or indoor worker (n = 1), homemaker (n = 5), retired (n = 3), unemployed (n = 4), and other (guard; n = 1).

Of 5 participants with evidence of recent exposure to CCHFV, 4 reported participating in >1 high-risk activity in the previous 4 months: 3 milked animals, 2 helped birth animals, 1 sheared animals, and 1 slaughtered animals. One participant reported experiencing an illness with joint pain in the previous 4 months. Three were from non–CCHF-endemic villages, which could suggest a wider range of virus circulation than previously thought.

In logistic regression, controlling for age and sex, participation in >1 high-risk activity had a statistically significant association with IgG or IgM seropositivity (adjusted OR [aOR] 5.6, 95% CI 1.1–29.7). Being >50 years of age was associated with having a history of infection but was not a risk factor for incident infection. Villages at lower elevations were more likely to have >1 person seropositive for CCHFV in logistic regression, but the association was not statistically significant (p = 0.41).

**Figure 2:**
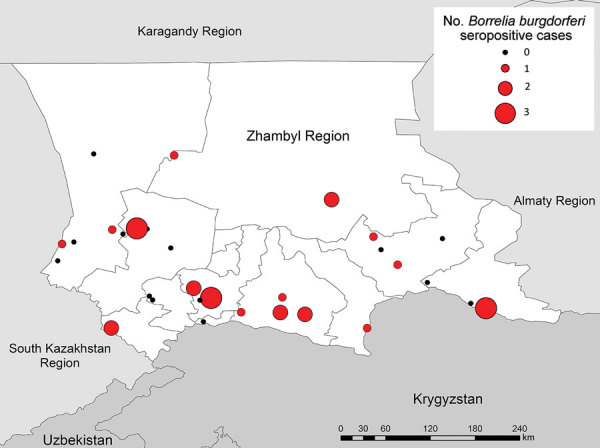
Number of *Borrelia burgdorferi*–seropositive cases in villages included in serologic survey for tickborne diseases, Zhambyl region, Kazakhstan. Circle size denotes the number of IgG antibody–positive serology results indicating past exposure to *B. burgdorferi.*

Of 911 participant samples tested for Lyme disease, 27 showed evidence of past exposure by IgG against tickborne borrelioses (Table 6). Weighted seroprevalence in the Zhambyl region was 2.4% (95% CI 1.2%–4.6%). We detected seropositive participants in 16/30 (53.3%) villages (Figure 2); occupations were farmer or herder (n = 5), gardener or fieldworker (n = 1), healthcare worker (n = 1), veterinarian (n = 1), office or indoor worker (n = 9), retired (n = 2), homemaker (n = 3), unemployed (n = 4), and other (geologist; n = 1) (Table 6). We did not identify specific activities statistically associated with seropositivity for Lyme disease, but we identified participants who were seropositive, even in a village at 2,590 m, an elevation at which the disease had not been reported in Kazakhstan.

**Figure 3:**
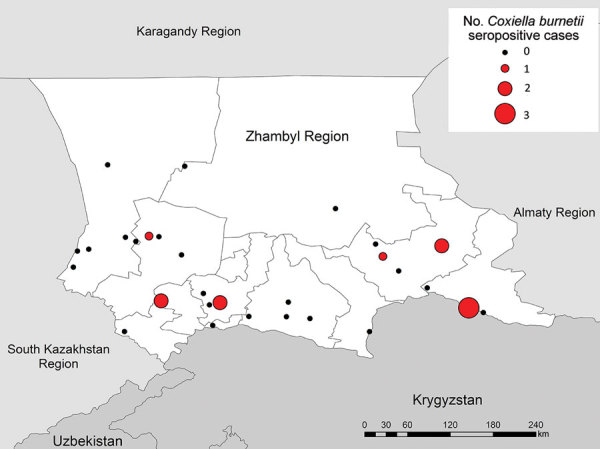
Number of *Coxiella burnetii*–seropositive cases in villages included in serologic survey for tickborne diseases, Zhambyl region, Kazakhstan. Circle size denotes the number of IgG antibody–positive serology results indicating past exposure to *C. burnetii.*

Of 910 samples tested for Q fever, 11 showed evidence of past exposure by *C. burnetii* IgG. Weighted seroprevalence was 1.3% (95% CI 0.3%–5.0%) with seropositivity in 5/30 (53.3%) villages (Figure 3). Occupations of the 11 seropositive participants were farmer or herder (n = 1), healthcare worker (n = 1), retired (n = 1), homemaker (n = 4), and inside or office worker (teacher, locksmith, or civil servant; n = 4;) (Table 6). Controlling for age and sex, history of herding (aOR 2.9, 95% CI 1.5–5.4) and slaughtering animals (aOR 2.7, 95% CI 1.5–4.8) had statistically significant associations with seropositivity. Villages at lower elevations were more likely to have >1 person seropositive for Q fever in logistic regression, but the association was not statistically significant (p = 0.49).

## Discussion

We conducted a serosurvey to update data on the prevalence of CCHF, Q fever, and Lyme disease in Kazakhstan. Because little is known about the seroprevalence of these diseases in central Asia, this study will increase regional awareness. Cases go undetected because of subclinical infections, nonspecific diagnostic methods, or poor surveillance. Our serosurvey identified persons exposed to these pathogens who might have been missed by existing surveillance platforms.

We found a weighted seroprevalence of 1.2% of CCHF in the study region, comparable to findings from studies in Turkey, Iran, and Bulgaria that reported seroprevalences of 2.3%–2.8% (*20*–*22*). We found a seroprevalence of 3.4% in villages classified as CCHF-endemic, similar to findings from studies in Bulgaria, China, Georgia, Kosovo, and Turkey that reported seroprevalences of 3%–4% (*16*,*22*–*28*). Most CCHFV serosurveys have been conducted in the Middle East, with a few in Asia, and prevalence estimates range widely, even in the same country. 

The comparability of our results to other published surveys is limited because many studies sampled during an outbreak or only sampled high-risk populations. Another CCHFV serosurvey from Kazakhstan found a seroprevalence of 12.7% among patients hospitalized with a fever of unknown origin in the Almaty and Kyzylorda regions (*29*). Studies of persons exposed to livestock in Iran and Turkey reported CCHFV seroprevalences >12% (*30*,*31*). Serosurveys in abattoir workers reported seroprevalences ranging from 0.75% to 16.5% (*32*,*33*). Studies in hyperendemic territories reported seroprevalences >10% in the general population (*34*–*41*), and a study in Kosovo reported 24% seroprevalence (*42*).

We found moderate seroprevalence (2.4%) for *B. burgdorferi* compared with findings for other countries in the region. For instance, a serosurvey in Ukraine found seroprevalences of 25%–38% in a healthy population depending on the ecologic zone (*43*). However, seroprevalence could be caused by other *Borrelia* species in that region and might not be specific to the Lyme disease group of *Borreliae*. In addition, <3 of 35 persons tested in some villages were seropositive (*43*).

We also found a lower weighted seroprevalence for Q fever (1.3%) than most reports. Our findings more closely approximate the 3.1% seroprevalence reported in the United States (*44*). However, as with CCHFV, prevalence of past infection varies widely by location. For instance, reports from Turkey demonstrate ≈4% seroprevalence in urban areas but 19% in rural areas (*45*). As we saw with Lyme disease, some villages in our sample had <3 of 35 participants who tested seropositive. Previous studies identified higher seroprevalence for Q fever in butchers (*46*,*47*), and our study showed 30.6% of participants seropositive for Q fever had butchered animals or handled raw meat.

A limitation of our study is that the Lyme disease assay was designed for broader reactivity and was not analytically specific to a single agent. This assay likely also reacts with relapsing fever *Borreliae*, which has unknown distribution in Kazakhstan. Further, validation studies from Vanhomwegen et al. (*17*) reported an analytic sensitivity of 80% for CCHF IgG Vector-Best kits and 88% for the IgM kits, with a specificity of 100% for both, so the true seroprevalence could be underestimated. The same is true for Lyme disease; a study reported a sensitivity of only 68.8% for the Vector-Best Lyme IgG kit (*18*).

We were surprised by the few reports of tick bites, considering that ≈90% of respondents listed ticks as a major problem and about one third had found ticks on their livestock. A previous survey identified crushing ticks with bare hands as common and a risk factor for CCHF (*16*). However, most respondents in our study reported crushing ticks with an object, suggesting contact with livestock could be a more common route of exposure among participants. This possibility could be problematic because <20% of respondents identified infected animals as a potential source of transmission. In addition, nearly half did not wear PPE when slaughtering animals, an exceptionally high-risk activity. The low recognition of the role of livestock in CCHF transmission is seen in other regions (*48*,*49*), but targeted educational campaigns have improved knowledge of transmission routes (*50*).

Our results have been translated into direct public health action. For instance, the serosurvey revealed that CCHFV is circulating in areas previously unknown to have CCHF activity. Because such areas were not prioritized for educational activities, knowledge of CCHF and modes of transmission was low compared with areas of known transmission. In addition, whereas the KAP/risk factor survey revealed that most respondents understood the risks posed by ticks and many took precautions against tick bites, most did not understand the role animals play in these zoonoses, nor did they wear proper PPE when performing high-risk activities. We helped the health department clarify their pamphlets to state specific high-risk activities and describe which PPE should be worn during each activity. Formative research into the availability and affordability of PPE, as well as the cultural perceptions of PPE when performing activities that may have ritualistic significance, such as slaughtering, is warranted.

A One Health approach that recognizes the interconnectedness of animal, human, and environmental health is needed for effective zoonotic and vectorborne disease control. This study incorporated personnel from the Kazakhstan Ministry of Health, Zhambyl Oblast Public Health Protection Department, and the Ministry of Agriculture. Additional studies in the region will analyze blood and ticks collected from livestock for evidence of past zoonotic infection. Combining the results of the human serosurvey with results of the animal and tick surveys will permit more in-depth investigations into the role of environmental factors, such as climate and elevation, in the transmission of these pathogens.
